# Renal pathology in adult and paediatric population of Japan: review of the Japan renal biopsy registry database from 2007 to 2017

**DOI:** 10.1007/s40620-023-01687-9

**Published:** 2023-08-19

**Authors:** Kazunori Goto, Takahiro Imaizumi, Riku Hamada, Kenji Ishikura, Tomoki Kosugi, Ichiei Narita, Hitoshi Sugiyama, Akira Shimizu, Hitoshi Yokoyama, Hiroshi Sato, Shoichi Mauryama

**Affiliations:** 1 Department of Nephrology, Nagoya University Graduate School of Medicine, Nagoya, Aichi Japan; 2 Center for Advanced Medicine and Clinical Research, Nagoya University Hospital, Nagoya, Aichi Japan; 3 Department of Nephrology, Tokyo Metropolitan Children’s Medical Center, Tokyo, Japan; 4 Department of Pediatrics, Kitasato University School of Medicine, Kanagawa, Japan; 5 Division of Clinical Nephrology and Rheumatology, Niigata University Graduate School of Medical and Dental Sciences, Niigata, Japan; 6 Department of Medicine, Kawasaki Medical School General Medical Center, Okayama, Japan; 7 Department of Medical Care Work, Kawasaki College of Allied Health Professions, Okayama, Japan; 8 Department of Analytic Human Pathology, Nippon Medical School, Tokyo, Japan; 9 Department of Nephrology, Kanazawa Medical University School of Medicine, Ishikawa, Japan; 10 JR Sendai Hospital, Miyagi, Japan

**Keywords:** Kidney biopsy, Renal pathology, Disease aetiology, Japan renal biopsy registry

## Abstract

**Background:**

The Japan Renal Biopsy Registry (J-RBR), a nationwide, web-based, registry system, started in 2007. This study aimed to summarise the epidemiology of biopsy-diagnosed kidney disease in Japan over 10 years.

**Methods:**

We analysed the J-RBR database, from 2007 to 2017. Patients’ clinical data collected at the time of biopsy and histopathological diagnoses were used for epidemiological and clinicopathologic analyses.

**Results:**

The predominant renal biopsy diagnoses were immunoglobulin A nephropathy (39.2%), lupus nephritis (6.5%) and minimal change disease (6.0%) in younger adults (19–64 years), and membranous nephropathy (17.4%), antineutrophil cytoplasmic antibody-associated vasculitis or anti-glomerular basement membrane glomerulonephritis (13.0%), and immunoglobulin A nephropathy (12.5%) in older adults (≥ 65 years). The percentages of patients diagnosed with membranoproliferative glomerulonephritis and immunoglobulin A nephropathy decreased, whereas those with immunoglobulin A vasculitis and diabetic nephropathy increased over the decade. In paediatric patients (< 19 years), immunoglobulin A nephropathy (36.1%), minimal change disease (17.6%), and immunoglobulin A vasculitis (8.6%) were the predominant diagnoses. The percentage of patients diagnosed with immunoglobulin A vasculitis increased over the decade. Based on the sex distribution, minimal change disease and membranous nephropathy were predominant in men aged < 20 and > 40 years, respectively, whereas immunoglobulin A vasculitis and antineutrophil cytoplasmic antibody-associated vasculitis or anti-glomerular basement membrane glomerulonephritis were predominant in women in their 20s and 30s and aged < 50 years, respectively. Immunoglobulin A nephropathy was predominant in men at most ages and in women in their 20s to 40s.

**Conclusions:**

This study describes the distribution and changes in kidney biopsy diagnoses over 10 years in Japan and paves the way for future research on kidney diseases in adults and children.

**Graphical abstract:**

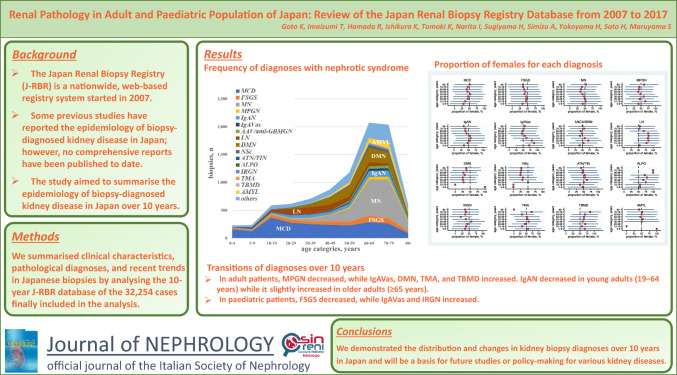

**Supplementary Information:**

The online version contains supplementary material available at 10.1007/s40620-023-01687-9.

## Introduction

Renal biopsy remains a crucial diagnostic tool for patients with kidney diseases or urinary abnormalities, including haematuria and proteinuria. Renal biopsy registries are, consequently, very useful in studying disease prevalence. Kidney disease diagnoses differ among countries; for example, focal segmental glomerular sclerosis (FSGS) and diabetic nephropathy (DMN) are major diagnoses in the USA and Canada, whereas immunoglobulin A nephropathy (IgAN) is a major diagnosis in Europe and Asia [1]. For the individual patient, clarifying the aetiology and pathophysiology is necessary for treatment decisions and estimation of prognosis [2], and clarifying the distribution of biopsy-diagnosed kidney disease is critical from a public health perspective; revealing the epidemiology of renal disease may contribute to determining the appropriate allocation of medical resources and reducing the number of patients with chronic kidney disease (CKD) or undergoing maintenance dialysis.

Japan’s first web-based nationwide kidney disease registration project includes the Renal Biopsy Registry (J-RBR: Japan Renal Biopsy Registry) and the registry of non-renal biopsy cases (J-KDR: Japan Kidney Disease Registry). The J-RBR was set up in July 2007, and its epidemiological data have substantiated several reports [3–6]. Under this registration system, each case diagnosis comprises three elements: clinical diagnosis, cause classification, and disease-type classification [3]. The J-RBR is the world’s largest registry of renal biopsy cases, with a cumulative total of 41,040 registered cases from 143 institutions nationwide, as of 15 January, 2018. In January 2018, the J-RBR system was revised; the diagnostic panel and clinical information items were updated [7].

In this report, we summarise the clinical characteristics, pathological diagnoses, and recent trends in Japanese biopsies, by analysing the 10-year J-RBR database of the 32,254 cases eventually included in the analysis.

## Materials and methods

### Registry system and patients

This cross-sectional study used data from patients recorded in the J-RBR from June 2007 to December 2017. Patient data, including age, sex, laboratory data, and clinical and pathological diagnoses, were recorded electronically at each institution and registered on the J-RBR web page. Patients were divided into the paediatric (< 19 years) and adult (≥ 19 years) groups. Furthermore, the adult group was analysed by subgroup as follows: younger adults (19–64 years) and older adults (≥ 65 years). Paediatric patients were stratified into subgroups by age (0–4, 5–9, 10–14, and 15–18 years) and analysed to compare with reports from other countries.

### Clinical and histopathological diagnoses and laboratory data

Basic demographic data included age, sex, and clinical diagnoses. Three classifications (clinical diagnosis, histological diagnosis by pathogenesis, and histological diagnosis by histopathology) were selected from the J-RBR for each case. Clinical diagnoses were classified as follows: acute nephritic syndrome, rapidly progressive nephritic syndrome, recurrent or persistent haematuria, chronic nephritic syndrome, nephrotic syndrome, renal disorder with metabolic disease, renal disorder with collagen disease or vasculitis, hypertensive nephropathy, inherited renal disease, acute renal failure, drug-induced nephropathy, renal transplantation, and others. Definitions of the first five clinical glomerular diseases were based on clinical syndromes and glomerular histopathology [8]. Renal histological diagnosis was classified according to pathogenesis or histopathology, as described in Supplementary Table 1. We extracted and separated diagnoses by pathogenesis and/or histopathology (Supplementary Table 1) for comparison with reports from other countries.

The following data were collected at the time of renal biopsy: serum creatinine concentration (sCr), 24-h proteinuria, red blood cells (RBCs) in the urine sediment, and presence of arterial hypertension (standard blood pressure value for each age) [9]. Proteinuria was evaluated by urine storage or spot urine. The estimated glomerular filtration rate (eGFR) was calculated by formulae for Japanese subjects, which were 194 × sCr^−1.094^ × age^−0.287^ (× 0.739 if female) for adult patients [10], and 110.2 × (reference sCr/sCr) + 2.93 (reference sCr values are calculated by body length) for paediatric patients [11]. Proteinuria and nephrotic-range proteinuria were defined as ≥ 0.3 g/day and ≥ 3.5 g/day, respectively, for adults, and ≥ 0.15 g/day and ≥ 2.0 g/day, respectively, for paediatric patients. Nephrotic syndrome was defined as a clinical diagnosis or laboratory data consistent with nephrotic syndrome, massive proteinuria (adults, ≥ 3.5 g/day; children, ≥ 2.0 g/day), and hypoalbuminemia (adults, < 3 g/dl; children, < 2.5 g/day). Nephritic syndrome was defined as non-nephrotic proteinuria with an eGFR < 60 ml/min/1.73 m^2^, haematuria (RBC ≥ 5 per high-power field), and arterial hypertension [12].

All participants provided written informed consent, and the study was approved by the ethics committees of the Japanese Society of Nephrology (JSN), Okayama University Graduate School of Medicine, Dentistry, and Pharmaceutical Sciences, and a local committee of participating centres and their affiliate hospitals; the study was carried out in accordance with the Helsinki Declaration. The J-RBR/J-KDR was registered in the Clinical Trial Registry of the University Hospital Medical Information Network (UMIN) (registration number UMIN000000618).

### Statistics

Continuous variables were expressed as mean ± standard deviation (SD). We used a chi-squared test to compare proportions of categorical variables among age groups. For the analysis of the trend in disease prevalence over the study period, we used the Cochran–Armitage test and logistic regression analysis (date of renal biopsy, age group, sex and interaction terms as explanatory variables). Sex differences in disease frequencies were expressed as the value of proportion of females (%) ± SD. *P* < 0.05 was considered statistically significant. Statistical analyses were conducted using Stata/SE (version 15.0; StataCorp LLC, College Station, TX, USA).

## Results

### Patient population and characteristics

Patient selection is shown in Supplementary Fig. 1A. Between 2007 and 2017, 41,040 renal biopsy records were referred to the J-RBR or J-KDR. A total of 8786 biopsy records were excluded from analysis. These included 4828 records registered to J-KDR or other registries, 2710 records identified as repeat biopsies (second or third), and 1248 biopsy records of kidney transplant recipients. Of the remaining 32,254 records, 3526 (10.9%) biopsies were performed in paediatric patients; 19,658 (60.9%) in younger adults (19–64 years); and 9070 (28.1%) in older adults (≥ 65 years). The annual total number of renal biopsies registered for J-RBR/J-KDR is shown in Supplementary Fig. 1B.

Clinical characteristics are shown in Table 1. In adult patients, the proportion of males was 51.0% in younger adults and 58.2% in older adults. The proportion of patients with nephrotic syndrome was 22.4% in younger adults and 41.6% in older adults. The proportion of patients with nephritic syndrome was 31.6% in younger adults and 25.2% in older adults. Paediatric patients were predominantly male in most age groups, accounting for 63.1% of children aged 0–4 years. Nephrotic syndrome was more common in younger age groups, accounting for 63.1% of patients aged 0–4 years, whereas nephritic syndrome was more common in the older paediatric age groups, accounting for 25.6% of patients aged 15–18 years.

**Table 1:** Clinical characteristics at the time of biopsy

	0–4 years (*N* = 371)	5–9 years (*N* = 727)	10–14 years (*N* = 920)	15–18 years (*N* = 1508)	19–64 years (*N* = 19,658)	65 ≤ years (*N* = 9070)
Age, y	2.9 ± 1.1	7.1 ± 1.4	12.2 ± 1.4	16.5 ± 1.1	43.7 ± 13.4	72.5 ± 5.5
Male/female	234/137	439/288	454/466	987/840	10,019/9639	5281/3789
Serum creatinine, mg/dl	0.4 ± 0.6	0.6 ± 1.1	0.7 ± 1.1	0.8 ± 0.8	1.2 ± 1.4	1.7 ± 1.7
Serum total protein, g/dl	5.7 ± 2.0	6.5 ± 2.7	6.7 ± 1.8	6.8 ± 2.3	6.8 ± 10.2	6.4 ± 7.9
Serum albumin, g/dl	4.4 ± 31.0	4.7 ± 22.8	4.1 ± 2.5	4.1 ± 2.9	3.8 ± 7.5	3.1 ± 3.9
Systolic blood pressure, mmHg	104 ± 11	105 ± 12	110 ± 12	115 ± 14	128 ± 20	136 ± 21
Diastolic blood pressure, mmHg	63 ± 11	63 ± 11	65 ± 11	67 ± 11	78 ± 14	76 ± 13
Hypertension	80 (21.6)	102 (14.0)	62 (6.7)	123 (8.2)	5,269 (26.8)	3,642 (40.2)
eGFR, ml/min/1.73 m^2^	118.5 ± 42.5	107.1 ± 33.8	109.1 ± 40.5	101.9 ± 33.3	67.4 ± 30.6	44.2 ± 24.3
eGFR 0–14 ml/min/1.73 m^2^	5 (1.3)	14 (1.9)	12 (1.3)	8 (0.5)	898 (4.6)	1082 (11.9)
eGFR 15–29 ml/min/1.73 m^2^	4 (1.1)	6 (0.8)	10 (1.1)	14 (0.9)	1384 (7.0)	1750 (19.3)
eGFR 30–44 ml/min/1.73 m^2^	6 (1.6)	10 (1.4)	13 (1.4)	20 (1.3)	2292 (11.7)	1980 (21.8)
eGFR 45–59 ml/min/1.73 m^2^	8 (2.2)	14 (1.9)	19 (2.1)	49 (3.2)	3335 (17.0)	1891 (20.9)
eGFR 60–89 ml/min/1.73 m^2^	33 (8.9)	70 (9.6)	81 (8.8)	309 (20.5)	7318 (37.2)	2031 (22.4)
eGFR ≥ 90 ml/min/1.73 m^2^	315 (84.9)	613 (84.3)	785 (85.3)	1,080 (71.6)	4362 (22.2)	336 (3.3)
Urine protein, g/day	2.9 ± 5.2	1.0 ± 2.4	1.0 ± 2.4	1.6 ± 3.1	2.1 ± 3.1	2.9 ± 3.3
Nephrotic range proteinuria	119 (32.1)	176 (24.2)	123 (13.4)	289 (19.2)	4468 (22.7)	3596 (39.7)
Hematuria (urine RBCs ≥ 5/HPF)	175 (47.2)	468 (64.4)	556 (60.4)	1,028 (68.2)	12278 (62.5)	5125 (56.5)
Nephrotic syndrome	234 (63.1)	236 (32.5)	226 (24.6)	292 (19.4)	4402 (22.4)	3770 (41.6)
Nephritic syndrome	10 (2.7)	66 (9.1)	128 (13.9)	386 (25.6)	6215 (31.6)	2283 (25.2)

Data are expressed mean ± SD or n (%)

*GFR* glomerular filtration rate, *Cr* creatinine, *RBCs* red blood cells, *HPF* high power field, Nephrotic-range proteinuria was defined as ≥ 3.5 g/day for adults (19 ≤ years), and ≥ 2.0 g/day for paediatric patients (< 19 years)

### Renal biopsy diagnoses distributions

Diagnoses distributions are shown in Tables 2 (adults) and 3 (paediatric patients) and Supplementary Fig. 2. The dominant renal biopsy diagnoses were IgAN (39.2%), lupus nephritis (LN) (6.5%), and minimal change disease (MCD) (6.0%) in younger adults and membranous nephropathy (MN) (17.4%), antineutrophil cytoplasmic antibody-associated vasculitis (AAV)/anti-glomerular basement membrane glomerulonephritis (anti-GBMGN) (13.0%), and IgAN (12.5%) in older adults; however, MCD, IgAN, and immunoglobulin A vasculitis (IgAVas) were dominant in paediatric patients. Minimal change disease and FSGS were more common in younger paediatric patients (0–4 years), and IgAN was more common in older paediatric patients (≥ 5 years).

**Table 2:** Distribution of renal biopsy diagnoses in adult patients

Diagnosis	Younger adults (19–64 years)	Older adults (≥ 65 years)	Total
MCD	1171 (6.0)	534 (5.9)	1705 (5.9)
FSGS	675 (3.4)	298 (3.3)	973 (3.4)
MN	1088 (5.5)	1578 (17.4)	2,666 (9.3)
MPGN	136 (0.7)	154 (1.7)	290 (1.0)
IgAN	7700 (39.2)	1,138 (12.5)	8838 (30.8)
IgAVas	532 (2.7)	198 (2.2)	730 (2.5)
AAV/anti-GBMGN	535 (2.7)	1,183 (13.0)	1718 (6.0)
LN	1268 (6.5)	148 (1.6)	1416 (4.9)
DMN	1028 (5.2)	726 (8.0)	1754 (6.1)
NSc	856 (4.4)	597 (6.6)	1453 (5.1)
ATN/TIN	576 (2.9)	455 (5.0)	1,031 (3.6)
ALPO	69 (0.4)	1 (0.0)	70 (0.2)
IRGN	176 (0.9)	128 (1.4)	304 (1.1)
TMA	85 (0.4)	35 (0.4)	120 (0.4)
TBMD	284 (1.4)	21 (0.2)	305 (1.1)
AMYL	151 (0.8)	273 (3.0)	424 (1.5)
Others	3328 (16.9)	1603 (17.7)	4931 (17.2)
Total	19,658 (100.0)	9070 (100.0)	28,728 (100.0)

Data are expressed as *n* (%)

*MCD* minimal change disease, *FSGS* focal segmental glomerulosclerosis, *MN* membranous nephropathy, *MPGN* membranoproliferative glomerulonephritis, *IgAN* IgA nephropathy, *IgAVas* IgA vasculitis, *AAV* antineutrophil cytoplasmic antibody-associated vasculitis, *anti-GBMGN* anti-glomerular basement membrane glomerulonephritis, *LN* lupus nephritis, *DMN* diabetic nephropathy, *NSc* nephrosclerosis, *ATN* acute tubular necrosis, *TIN* tubulointerstitial nephritis, *ALPO* Alport syndrome, *IRGN* infection-related glomerulonephritis, *TMA* thrombotic microangiopathy, *TBMD* thin basement membrane disease; AMYL amyloidosis

**Table 3:** Distribution of renal biopsy diagnoses in different age categories in paediatric patients

Diagnosis	0–4 years	5–9 years	10–14 years	15–18 years	Total
MCD	161 (43.4)	128 (17.6)	138 (15.0)	193 (12.8)	620 (17.6)
FSGS	34 (9.2)	23 (3.2)	28 (3.0)	44 (2.9)	129 (3.7)
MN	8 (2.2)	25 (3.4)	20 (2.2)	18 (1.2)	71 (2.0)
MPGN	6 (1.6)	11 (1.5)	25 (2.7)	13 (0.9)	55 (1.6)
IgAN	28 (7.5)	199 (27.4)	327 (35.5)	719 (47.7)	1273 (36.1)
IgAVas	29 (7.8)	160 (22.0)	68 (7.4)	45 (3.0)	302 (8.6)
AAV/anti-GBMGN	1 (0.3)	6 (0.8)	15 (1.6)	9 (0.6)	31 (0.9)
LN	3 (0.8)	10 (1.4)	62 (6.7)	85 (5.6)	160 (4.5)
DMN	0 (0.0)	1 (0.1)	1 (0.1)	0 (0.0)	2 (0.1)
NSc	1 (0.3)	0 (0.0)	1 (0.1)	5 (0.3)	7 (0.2)
ATN/TIN	4 (1.1)	11 (1.5)	19 (2.1)	24 (1.6)	58 (1.6)
ALPO	18 (4.9)	20 (2.8)	13 (1.4)	12 (0.8)	63 (1.8)
IRGN	2 (0.5)	12 (1.7)	7 (0.8)	9 (0.6)	30 (0.9)
TMA	5 (1.3)	2 (0.3)	1 (0.1)	2 (0.1)	10 (0.3)
TBMD	0 (0.0)	3 (0.4)	13 (1.4)	29 (1.9)	45 (1.3)
AMYL	1 (0.3)	0 (0.0)	1 (0.1)	0 (0.0)	2 (0.1)
Others	70 (18.9)	116 (16.0)	181 (19.7)	301 (20.0)	668 (18.9)
Total	371 (100.0)	727 (100.0)	920 (100.0)	1508 (100.0)	3526 (100.0)

Data are expressed as *n* (%)

*MCD* minimal change disease, *FSGS* focal segmental glomerulosclerosis, *MN* membranous nephropathy, *MPGN* membranoproliferative glomerulonephritis, *IgAN* IgA nephropathy, *IgAVas* IgA vasculitis, *AAV* antineutrophil cytoplasmic antibody-associated vasculitis, *anti-GBMGN* anti-glomerular basement membrane glomerulonephritis, *LN* lupus nephritis, *DMN* diabetic nephropathy, *NSc* nephrosclerosis, *ATN* acute tubular necrosis, *TIN* tubulointerstitial nephritis, *ALPO* Alport syndrome, *IRGN* infection-related glomerulonephritis, *TMA* thrombotic microangiopathy, *TBMD* thin basement membrane disease, *AMYL* amyloidosis

Distributions for every 15 years of age are shown in Supplementary Table 2. Most of the adolescent and young adult generation had IgAN. immunoglobulin A vasculitis was seen in approximately 12% of patients < 14 years old; however, it was seen in approximately 2–3% of the adolescent and young adult and older generations. Minimal change disease (9.8%) and LN (7.7%) were predominant in the adolescent and young adult generation.

### Proportion of females in each diagnosis category

**Fig. 1:**
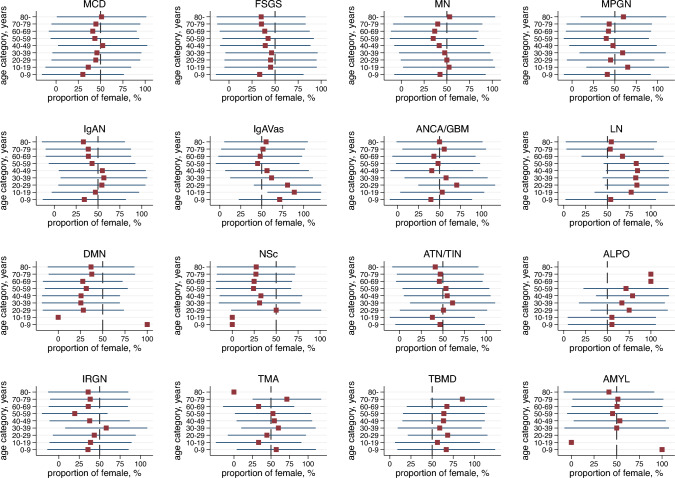
The proportion of females for each diagnosis. The horizontal bars show standard deviation. *MCD* minimal change disease, *FSGS* focal segmental glomerulosclerosis, *MN* membranous nephropathy, *MPGN* membranoproliferative glomerulonephritis, *IgAN* IgA nephropathy, *IgAVas* IgA vasculitis, *AAV* antineutrophil cytoplasmic antibody-associated vasculitis, *anti-GBMGN* anti-glomerular basement membrane glomerulonephritis, *LN* lupus nephritis, *DMN* diabetic nephropathy, *NSc* nephrosclerosis, *ATN* acute tubular necrosis, *TIN* tubulointerstitial nephritis, *ALPO* Alport syndrome, *IRGN* infection-related glomerulonephritis, *TMA* thrombotic microangiopathy, *TBMD* thin basement membrane disease, *AMYL* amyloidosis

We analysed the sex distribution in each disease across different age groups as shown in Fig. 1, by group of adults and paediatric patients, and total population as shown in Supplementary Table 3. Minimal change disease was more common in boys and slightly more common in men. Focal segmental glomerularsclerosis was more common in males of all ages. Membranous nephropathy was more common in males > 40 years old. Immunoglobulin A nephropathy was more common in males < 10 and > 50 years old and more often diagnosed in females in their 20–40s. Immunoglobulin A vasculitis had an equal percentage of males and females in the total population, but was more common in males especially < 10 years old and in females in their 20–30s. Antibody-associated vasculitis/anti-glomerular basement membrane glomerulonephritis was more common in females < 50 years old. Lupus nephritis was predominant in women of most ages. Diabetic nephropathy and nephrosclerosis (NSc) were mainly observed in adult patients and most commonly in adult males. Alport syndrome, and thin basement membrane disease were more common in women, while infection-related glomerulonephritis was more common in men.

### Diagnoses in nephrotic syndrome

The distributions of diagnoses are shown in Supplementary Tables 4A (adults) and 4B (paediatric patients) and Supplementary Fig. 3. In adult patients, MCD (26.6%), MN (16.2%), DMN (11.7%), and LN (10.5%) were the most common diseases, with the highest diagnosis rates in younger adults, whereas MN (32.5%), MCD (14.2%), DMN (9.6%), and amyloidosis (5.5%) were highest among older adults. Diabetic nephropathy was more common in younger than in older adults. In paediatric patients, MCD was the most common diagnosis, followed by IgAN, IgAVas, and LN.

### Nephrotic syndrome diagnoses distribution

**Fig. 2:**
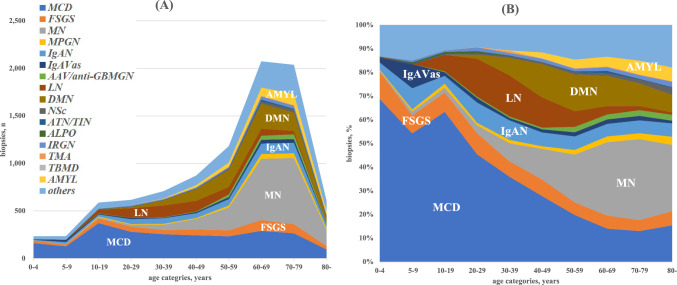
The frequency of diagnoses with nephrotic syndrome for each 10-year age group. **A** The number and **B** percentage of diagnoses are shown. Data for each panel are shown in Supplementary Table 5

The distributions of nephrotic syndrome diagnoses by age category are shown in Fig. 2 and Supplementary Table 5. Nephrotic syndrome biopsies were performed most frequently in patients 60–69 years of age with MN (31.1%), MCD (14.0%), and DMN (12.8%). Lupus nephritis was most frequently diagnosed in patients aged 20–29 and 30–39 years (16.0% and 17.3%, respectively). Minimal change disease was the most common diagnosis for paediatric patients with nephrotic syndrome.

### Transitions of diagnoses over 10 years

Transitions of diagnoses are shown in Supplementary Tables 6A (adults) and 6B (paediatric patients). Among all adult patients, the results of the analysis of trend showed that the proportion of patients diagnosed with membranoproliferative glomerulonephritis (MPGN) and IgAN decreased over 10 years, whereas those diagnosed with IgAVas, DMN, thrombotic microangiopathy, and thin basement membrane disease increased. Logistic regression analysis, including age-by-time (year) of renal biopsy as explanatory variables, showed that IgAN decreased in young adults (19–64 years), whereas it slightly increased in older adults (≥ 65 years). In paediatric patients, the proportion of patients diagnosed with FSGS decreased over 10 years, whereas those diagnosed with IgAVas and infection-related glomerulonephritis increased.

### Ten-year urine abnormality distributions

Transitions of 10-year urine abnormality distributions are shown in Supplementary Fig. 4. The proportions of haematuria alone and negative urinary findings were higher in paediatric patients than in adults. The proportion of patients with haematuria alone or without urinary abnormalities decreased in both adult and paediatric patients, while the proportion of patients with proteinuria alone or proteinuria/haematuria increased in adult patients.

### Nephritic syndrome diagnosis distributions

The distributions of nephritic syndrome diagnoses are shown in Supplementary Table 7. Immunoglobulin A nephropathy was more common in both adults (43.1%) and paediatric patients (63.6%) with nephritic syndrome than in all patients. The proportion of AAV/anti-GBMGN was high in adults (8.0%), whereas the proportion of IgAVas was high in paediatric patients (6.4%).

## Discussion

With 143 participating institutions, the J-RBR is one of the largest renal biopsy registries worldwide. It includes a total of 41,040 cases and covers major renal diseases affecting children and adults. This is the first report of a total list of renal biopsies performed in Japan over a period of 10 years (2007–2017). This comprehensive report provides epidemiological descriptions and clinicopathological correlations for kidney disease among all individuals who underwent kidney biopsy.

According to a previous report [1], FSGS and DMN were the main diagnoses in the US and Canada, and IgAN was the main diagnosis in Europe and Asia. This study showed that the most common renal biopsy diagnoses in all adults were IgAN and MN, which was consistent with reports from several European registries [13–16]. Immunoglobulin A nephropathy is the most common diagnosis among younger adults, and MN is the most common in older adults, which is consistent with a previous report from Japan [5]. Amyloidosis, AAV/anti-GBMGN, and DMN were more common in older adults, which is consistent with reports from several studies of older cohorts [8, 17–20]. In paediatric patients, the most common diagnosis was IgAN (36.1%), followed by MCD (17.6%) and IgAVas (8.6%). Furthermore, the proportion of patients with MCD as the main diagnosis of nephrotic syndrome was higher in the younger population, whereas the proportion with IgAN as the main diagnosis of nephritic syndrome was higher in the older population. Reports from the Czech Republic [16], Italy [21], and Korea [22] showed that IgAN was the most common diagnosis, followed by MCD, whereas other reports from China [23] and India [24] showed that MCD was the most common diagnosis, followed by IgAN. The proportion of IgAN in paediatric patients was higher than in other Asian countries. One possible reason is that public medical administration policies in Japan allow free screening for urinary abnormalities at least once yearly for all ages; thus, more patients in Japan undergo renal biopsy due to abnormal urinalysis without other symptoms, such as proteinuria/haematuria, than in other countries. While Krogsbøll et al. [25] reported that general health checks did not reduce morbidity or mortality, either overall or for cardiovascular- or cancer-related causes, a study from Japan [26] reported that those who have not undergone screening have a high risk of end-stage kidney disease. Other studies [27, 28] have further reported that school urinalysis examinations significantly improve chronic glomerulonephritis prognoses.

A previous report in Japan [5] demonstrated that the most common clinical diagnosis in adults was nephritic syndrome, followed by nephrotic syndrome, which is consistent with our findings. Our results demonstrated that nephrotic syndrome was more common in older adults, and nephritic syndrome was more common in younger adults. Among older adults, the proportion diagnosed with nephrotic syndrome was approximately 40%, which is consistent with reports indicating that nephrotic syndrome is the leading clinical syndrome in patients aged ≥ 65 years who underwent native kidney biopsy [13, 17, 20, 29, 30]. Among all adults (≥ 19 years) with nephrotic syndrome, the most common diagnosis was MN, followed by MCD and DMN, which was slightly different from reports in China (MN, followed by MCD and IgAN) [31], Poland (MN, followed by amyloidosis and FSGS) [12], and the Czech Republic (MCD, followed by IgAN and MN) [16]. In the paediatric population, the highest proportion of patients diagnosed with nephrotic syndrome were those 0–4 years old. The proportion diagnosed with MCD was > 60%, which was much higher than that in other Asian countries, such as China (47–54%) [23] and India (32%) [25].

We compared frequencies of diagnoses by sex and found that among all individuals who underwent kidney biopsy, NSc and DMN were diagnosed more frequently in males, whereas LN and thin basement membrane disease were diagnosed more frequently in females; these sex distributions were similar to reports from the US and Europe [1, 12, 16]. Several of our findings have previously been reported; the differences of these reports were partly due to race and threshold for indications of renal biopsy. We found that IgAN and IgAVas were diagnosed more frequently in females but were a common diagnosis in males in the sum of cohorts from North America, Europe, Latin America, and Asia [1]. Furthermore, we investigated sex differences in disease frequencies stratified by age categories. Immunoglobulin A nephropathy, as well as IgAVas, was diagnosed frequently in males < 10 years old. Minimal change disease was diagnosed more frequently in boys, consistent with a report from China [23]. Thus, paediatric males have a higher risk of major glomerular diseases, such as MCD, IgAN, and IgAVas. A previous report [1] showed that MN was common in males, predominantly those aged 40–80 years. In a previous report from Japan [32], childhood-onset AAV was predominantly found in females and frequently detected in school urinary screenings, which supports our result of a female predominance of AAV/anti-GBMGN in the young population, aged 10–30 years. Lupus nephritis is a common disease in females, and the results of this study confirmed that LN was more often diagnosed by renal biopsy in females. Diabetic nephropathy and NSc were diagnosed more frequently in males, which was consistent with a previous report [1]. Alport syndrome was more often diagnosed by renal biopsy in females, and a possible reason for this was that most cases of Alport syndrome are the X-linked dominant inherited type [33], and severe symptoms are seen in male patients. However, more female patients with mild symptoms are likely to undergo renal biopsy. This is the first report showing comprehensive data on the sex distribution of renal biopsy diagnoses across all ages.

This study demonstrates changes in distribution patterns of diagnoses over 10 years. In China, the proportion of MN in adults has been increasing, possibly due to environmental factors such as air pollution [31]. The US demonstrated increasing proportions of FSGS and DMN diagnoses, possibly due to lifestyle factors such as obesity and diabetes mellitus [34]. In adult and paediatric patients, the proportions of patients diagnosed with MCD and MN were stable over time. The proportion of AAV/anti-GBMGN was also stable, which was consistent with reports from China [31] and the USA [34]. However, the reasons for these results could not be determined from this epidemiological study. In adult patients, the proportion of patients diagnosed with DMN increased, which was consistent with reports from China [31] and the USA [34], whereas that with NSc was stable over 10 years. One possible reason is that the number of patients with hypertension and/or diabetes mellitus has recently increased, and renal biopsy is recommended for patients with proteinuria, which is more likely to appear in patients with DMN than in those with NSc. The proportion of patients diagnosed with MPGN decreased during the study period. Membranoproliferative glomerulonephritis is diagnosed by an injury pattern, and it includes various diseases such as primary MPGN and C3 nephropathy. It is possible that C3 nephropathy may have been gradually registered as an independent category from MPGN [35]. The proportion of patients with an IgAN diagnosis decreased in adults for the study duration, whereas the frequency of IgAN diagnoses was stable in the USA [34] and increased in Germany [36]. A potential reason is that renal biopsies for patients with absent urinary findings tended to be restrained over the decade, as shown in Supplementary Figure 4. The indications for renal biopsy changed to focus on proteinuria over the 10-year period, and cases with only haematuria were unlikely to undergo renal biopsy. In paediatric patients who underwent kidney biopsy, IgAVas diagnosis was found to be increasing. The reason for this was not clear. However, a report from China [31] including both adult and paediatric patients showed that IgAVas as well as IgAN, was gradually decreasing.

Our study has several limitations. First, we were unable to count the total incidence rate of renal biopsy in Japan, as only a portion of all renal biopsy facilities participated in J-RBR. According to past surveys, approximately 20,000 renal biopsies are performed annually in Japan, and J-RBR covered approximately 20% of these cases. Second, the J-RBR records three diagnoses (clinical diagnosis, histological diagnosis by pathogenesis, and histological diagnosis by histopathology) for each case. Inconsistencies may occur in the case records because we followed the glomerular disease classification originally proposed by the World Health Organization (WHO) in the 1980s [37] and revised in the 1990s [38], which described histopathological patterns of glomerular injury but did not encompass its aetiology. Third, the registration system was revised in 2018; some renal biopsy cases performed from 2016 to 2017 were registered in the revised J-RBR/J-KDR system and were not included in this study. Further analysis is warranted to determine the change in disease trends over longer periods. Fourth, the annual trend data were analysed without age adjustments. Japan’s population is ageing, and the proportion of older patients is on the rise, as shown in Supplementary Fig. 5; the age-dependent disease distribution may affect the annual transition data. Age-adjusted analysis is required to compensate for age-related demographic changes. Fifth, the time needed to confirm the diagnosis by renal biopsy varies from case to case due to the clinical course, general condition, and comorbidities, but the time from onset of urinary abnormalities to renal biopsy could not be analysed in this study due to lack of data.

In conclusion, this study showed pathological and clinical data from the renal biopsy registry over 10 years, which provided us with the prevalence and trends in the diagnoses of renal disease in Japan. The comprehensive data obtained in this study should serve as a basis for future studies on various renal diseases.

### Supplementary Information

Below is the link to the electronic supplementary material.

Supplementary file1 (DOCX 621 kb)

## Data Availability

The datasets generated and analysed during the current study are not publicly available because the consent obtained from the participants does not cover unlimited public sharing of the data but are available upon reasonable request.
