# Supplementary material for: Renal pathology in adult and paediatric population of Japan: review of the Japan renal biopsy registry database from 2007 to 2017
Source: J Nephrol. 2023 Aug 19;36(8):2257–67. doi: 10.1007/s40620-023-01687-9 (PMC10638177; doi:10.1007/s40620-023-01687-9)
Supplement: Supplementary file 1 — Supplementary file1 (DOCX 621 kb) [file 40620_2023_1687_MOESM1_ESM.docx]

**SUPPLEMENTARY DATA**

**Page 2 Supplementary Table 1.**

The list of kidney biopsy diagnoses in this study

**Page 3 Supplementary Table 2.**

Distribution of renal biopsy diagnoses by different age groups

**Page 4 Supplementary Table 3.**

Distribution of renal biopsy diagnoses by sex

**Page 5 Supplementary Table 4 A.**

Distribution of renal biopsy diagnoses of nephrotic syndrome in adult patients

**Page 6 Supplementary Table 4 B.**

Distribution of renal biopsy diagnoses of nephrotic syndrome in paediatric patients

**Page 7 Supplementary Table 5.**

Distribution of renal biopsy diagnoses of nephrotic syndrome by different age groups

**Page 8 Supplementary Table 6 A.**

Transitions of diagnoses over 10 years in adult patients

**Page 9 Supplementary Table 6 B.**

Transitions of diagnoses over 10 years in paediatric patients

**Page 10 Supplementary Table 7.**

Distribution of renal biopsy diagnoses of nephritic syndrome

**Page 11 Supplementary Figure 1 A.**

Flow of patient selection in the present study

**Supplementary Figure 1 B.**

Annual number of renal biopsies registered to J-RBR/J-KDR

**Page 12 Supplementary Figure 2.**

The frequency of total diagnoses by different age groups

**Page 13 Supplementary Figure 3.**

The frequency of diagnoses with nephrotic syndrome by different age groups

**Page 14 Supplementary Figure 4.**

The distribution of urine abnormalities over 10 years

**Page 15 Supplementary Figure 5.**

The distribution of age categories for a period of 10 years

**Page 16−21 Supplemental Note.**

The Investigators and Institutions Participating in the Japan Renal Biopsy Registry

**Supplementary Table 1. The list of kidney biopsy diagnoses in this study**

| **Diagnosis** | **(A) Pathogenesis (cause classification)** | **(B) Histopathology (disease type classification)** |
| --- | --- | --- |
| IgA nephritis (IgAN) | IgA nephritis |  |
| ANCA-associated vasculitis (AAV)/  anti-GBM glomerulonephritis (anti-GBMGN) | MPO-ANCA-positive nephritis |  |
|  | PR3-ANCA-positive nephritis |  |
|  | anti-GBM antibody nephritis |  |
| others^a^ | primary glomerular disease | minor glomerular abnormalities (MGA) |
| minimal change disease (MCD)^b^ |  |  |
| focal and segmental glomerulosclerosis (FSGS)^c^ |  | focal and segmental glomerulosclerosis |
| membranous nephropathy (MN) |  | membranous nephropathy |
| membranoproliferative glomerulonephritis (MPGN) |  | membranoproliferative glomerulonephritis (type I and III) |
| others |  |  |
|  | not diagnosed |  |
|  |  |  |
| acute tubular necrosis (ATN)/  tubulointerstitial nephritis (TIN) |  | acute tubular necrosis |
|  |  | acute interstitial nephritis |
|  |  | chronic interstitial nephritis |
| amyloid nephropathy (AMYL) | amyloid nephropathy |  |
| Alport syndrome (ALPO) | Alport syndrome |  |
| lupus nephritis (LN) | lupus nephritis |  |
| infection-related glomerulonephritis (IRGN) | infection-related glomerulonephritis |  |
| *excluded in this study*^d^ | transplanted kidney |  |
| diabetic nephropathy (DMN) | diabetic nephropathy |  |
| IgA vasculitis (IgAVas) | IgA vasculitis |  |
| thin basement membrane disease (TBMD) | thin basement membrane disease |  |
| thrombotic microangiopathy (TMA) | thrombotic microangiopathy |  |
| hypertensive nephrosclerosis (NSc) | hypertensive nephrosclerosis |  |

Diagnoses were defined by two classifications, the (A) pathogenesis and (B) histopathology. IgA, immunoglobulin A; MPO, myeloperoxidase; PR3, proteinase3; ANCA, anti-neutrophil cytoplasmic antibody; GBM, glomerular basement membrane

a MGA without clinical diagnosis of nephrotic syndrome was categorized as ‘others’.

b MCD was defined as MGA accompanied by nephrotic syndrome.

c the cause of FSGS included both primary and secondary.

d the transplanted kidney was excluded in this study.

**Supplementary Table 2. Distribution of renal biopsy diagnoses by different age groups**

| Diagnosis | 0–14 years | 15–29 years | 30–44 years | 45–59 years | ≥ 60 years |
| --- | --- | --- | --- | --- | --- |
| MCD | 427 (21.2) | 515 (9.8) | 392 (6.3) | 340 (5.2) | 651 (5.3) |
| FSGS | 85 (4.2) | 165 (3.1) | 211 (3.4) | 242 (3.7) | 399 (3.3) |
| MN | 53 (2.6) | 60 (1.1) | 174 (2.8) | 457 (7.0) | 1,993 (16.3) |
| MPGN | 42 (2.1) | 35 (0.7) | 35 (0.6) | 45 (0.7) | 188 (1.5) |
| IgAN | 554 (27.5) | 2,715 (51.7) | 2,907 (46.7) | 2,085 (31.8) | 1,850 (15.2) |
| IgAVas | 257 (12.7) | 163 (3.1) | 180 (2.9) | 142 (2.2) | 290 (2.4) |
| AAV/anti-GBMGN | 22 (1.1) | 38 (0.7) | 56 (0.9) | 235 (3.6) | 1,398 (11.5) |
| LN | 75 (3.7) | 405 (7.7) | 520 (8.4) | 331 (5.0) | 245 (2.0) |
| DMN | 2 (0.1) | 20 (0.4) | 211 (3.4) | 495 (7.5) | 1,028 (8.4) |
| NSc | 2 (0.1) | 44 (0.8) | 209 (3.4) | 401 (6.1) | 804 (6.6) |
| ATN/TIN | 34 (1.7) | 99 (1.9) | 131 (2.1) | 224 (3.4) | 601 (4.9) |
| ALPO | 51 (2.5) | 40 (0.8) | 22 (0.4) | 16 (0.2) | 4 (0.0) |
| IRGN | 21 (1.0) | 34 (0.6) | 50 (0.8) | 67 (1.0) | 162 (1.3) |
| TMA | 8 (0.4) | 11 (0.2) | 30 (0.5) | 31 (0.5) | 50 (0.4) |
| TBMD | 16 (0.8) | 76 (1.4) | 96 (1.5) | 112 (1.7) | 50 (0.4) |
| AMYL | 2 (0.1) | 0 (0.0) | 19 (0.3) | 81 (1.2) | 324 (2.7) |
| others | 367 (18.2) | 835 (15.9) | 978 (15.7) | 1,258 (19.2) | 2,161 (17.7) |
| total | 2,018 (100.0) | 5,255 (100.0) | 6,221 (100.0) | 6,562 (100.0) | 12,198 (100.0) |

Data are expressed as n (%). MCD, minimal change disease; FSGS, focal segmental glomerulosclerosis; MN, membranous nephropathy; MPGN, membranoproliferative glomerulonephritis; IgAN, IgA nephropathy; IgAVas, IgA vasculitis; AAV, antineutrophil cytoplasmic antibody-associated vasculitis; anti-GBMGN, anti-glomerular basement membrane glomerulonephritis; LN, lupus nephritis; DMN, diabetic nephropathy; NSc, nephrosclerosis; ATN, acute tubular necrosis; TIN, tubulointerstitial nephritis; ALPO, Alport syndrome; IRGN, infection-related glomerulonephritis; TMA, thrombotic microangiopathy; TBMD, thin basement membrane disease; AMYL, amyloidosis

**Supplementary Table 3. Distribution of renal biopsy diagnoses by sex**

| Diagnosis | Paediatiric patients  (< 19 years) | | Adult patients  (≥ 19 years) | | Total | |
| --- | --- | --- | --- | --- | --- | --- |
|  | Male/Female | Female (%) | Male/Female | Female (%) | Male/Female | Female (%) |
| MCD | 411/209 | (33.7) | 934/771 | (45.2) | 1345/980 | (42.2) |
| FSGS | 78/51 | (39.5) | 578/395 | (40.6) | 656/446 | (40.5) |
| MN | 38/33 | (46.5) | 1609/1057 | (39.6) | 1647/1090 | (39.8) |
| MPGN | 23/32 | (58.2) | 157/133 | (45.9) | 180/165 | (47.8) |
| IgAN | 708/565 | (44.4) | 4418/4420 | (50.0) | 5126/4985 | (49.3) |
| IgAVas | 167/135 | (44.7) | 349/381 | (52.2) | 516/516 | (50.0) |
| AAV/anti-GBMGN | 5/26 | (83.9) | 840/878 | (51.1) | 845/904 | (51.7) |
| LN | 36/124 | (77.5) | 289/1127 | (79.6) | 325/1251 | (79.4) |
| DMN | 2/0 | (0.0) | 1281/473 | (27.0) | 1283/473 | (26.9) |
| NSc | 6/1 | (14.3) | 1008/445 | (30.6) | 1014/446 | (30.5) |
| ATN/TIN | 34/24 | (41.4) | 521/510 | (49.5) | 555/534 | (49.0) |
| ALPO | 27/36 | (57.1) | 19/51 | (72.9) | 46/87 | (65.4) |
| IRGN | 18/12 | (40.0) | 191/113 | (37.2) | 209/125 | (37.4) |
| TMA | 5/5 | (50.0) | 58/62 | (51.7) | 63/67 | (51.5) |
| TBMD | 20/25 | (55.6) | 109/196 | (64.3) | 129/221 | (63.1) |
| AMYL | 1/1 | (50.0) | 215/209 | (49.3) | 216/210 | (49.3) |
| others | 364/304 | (45.5) | 2724/2207 | (44.8) | 3088/2511 | (44.8) |
| Total | 1943/1583 | (44.9) | 15300/13428 | (46.7) | 17243/15011 | (46.5) |

Note: data are expressed n (%). MCD minimal change disease; FSGS, focal segmental glomerulosclerosis; MN, membranous glomerulonephritis; MPGN, membranoproliferative glomerulonephritis; IgAN, IgA nephropathy; IgAVas, IgA vasculitis; AAV, antineutrophil cytoplasmic antibody associated vasculitis; anti-GBMGN, anti-glomerular basement membrane glomerulonephritis; LN, lupus nephropathy; DMN, diabetic mellitus nephropathy; NSc, nephrosclerosis; ATN, acute tubular necrosis; TIN, tubulointerstitial nephritis; ALPO, Alport syndrome; IRGN, infection related glomerulonephritis; TMA, thrombotic microangiopathy; TBMD, thin basement membrane disease; AMYL, amyloidosis

**Supplementary Table 4 A. Distribution of renal biopsy diagnoses of nephrotic syndrome in adult patients**

| Diagnosis | Nephrotic syndrome | | |
| --- | --- | --- | --- |
|  | Younger adults (19–64 years) | Older adults (≥ 65 years) | Total (≥ 19 years) |
| MCD | 1,171 (26.6) | 534 (14.2) | 1,705 (20.9) |
| FSGS | 281 (6.4) | 194 (5.1) | 475 (5.8) |
| MN | 715 (16.2) | 1,227 (32.5) | 1,942 (23.8) |
| MPGN | 68 (1.5) | 101 (2.7) | 169 (2.1) |
| IgAN | 292 (6.6) | 197 (5.2) | 489 (6.0) |
| IgAVas | 73 (1.7) | 65 (1.7) | 138 (1.7) |
| AAV/anti-GBMGN | 62 (1.4) | 85 (2.3) | 147 (1.8) |
| LN | 461 (10.5) | 69 (1.8) | 530 (6.5) |
| DMN | 514 (11.7) | 361 (9.6) | 875 (10.7) |
| NSc | 27 (0.6) | 70 (1.9) | 97 (1.2) |
| ATN/TIN | 17 (0.4) | 20 (0.5) | 37 (0.5) |
| ALPO | 8 (0.2) | 1 (0.0) | 9 (0.1) |
| IRGN | 59 (1.3) | 63 (1.7) | 122 (1.5) |
| TMA | 13 (0.3) | 8 (0.2) | 21 (0.3) |
| TBMD | 3 (0.1) | 1 (0.0) | 4 (0.0) |
| AMYL | 98 (2.2) | 208 (5.5) | 306 (3.7) |
| others | 540 (12.3) | 566 (15.0) | 1,106 (13.5) |
| total | 4,402 (100.0) | 3,770 (100.0) | 8,172 (100.0) |

Data are expressed as n (%). MCD, minimal change disease; FSGS, focal segmental glomerulosclerosis; MN, membranous nephropathy; MPGN, membranoproliferative glomerulonephritis; IgAN, IgA nephropathy; IgAVas, IgA vasculitis; AAV, antineutrophil cytoplasmic antibody-associated vasculitis; anti-GBMGN, anti-glomerular basement membrane glomerulonephritis; LN, lupus nephritis; DMN, diabetic nephropathy; NSc, nephrosclerosis; ATN, acute tubular necrosis; TIN, tubulointerstitial nephritis; ALPO, Alport syndrome; IRGN, infection-related glomerulonephritis; TMA, thrombotic microangiopathy; TBMD, thin basement membrane disease; AMYL, amyloidosis

**Supplementary Table 4 B. Distribution of renal biopsy diagnoses of nephrotic syndrome in paediatric patients**

| Diagnosis | Nephrotic syndrome | | |
| --- | --- | --- | --- |
|  | 0–4 years | 5–18 years | Total (≤ 18 years) |
| MCD | 161 (68.8) | 459 (60.9) | 620 (62.8) |
| FSGS | 27 (11.5) | 60 (8.0) | 87 (8.8) |
| MN | 1 (0.4) | 13 (1.7) | 14 (1.4) |
| MPGN | 1 (0.4) | 11 (1.5) | 12 (1.2) |
| IgAN | 7 (3.0) | 37 (4.9) | 44 (4.5) |
| IgAVas | 5 (2.1) | 33 (4.4) | 38 (3.8) |
| AAV/anti-GBMGN | 0 (0.0) | 2 (0.3) | 2 (0.2) |
| LN | 1 (0.4) | 36 (4.8) | 37 (3.7) |
| DMN | 0 (0.0) | 0 (0.0) | 0 (0.0) |
| NSc | 0 (0.0) | 2 (0.3) | 2 (0.2) |
| ATN/TIN | 0 (0.0) | 2 (0.3) | 2 (0.2) |
| ALPO | 0 (0.0) | 4 (0.5) | 4 (0.4) |
| IRGN | 0 (0.0) | 5 (0.7) | 5 (0.5) |
| TMA | 0 (0.0) | 0 (0.0) | 0 (0.0) |
| TBMD | 0 (0.0) | 0 (0.0) | 0 (0.0) |
| AMYL | 0 (0.0) | 0 (0.0) | 0 (0.0) |
| others | 31 (13.2) | 90 (11.9) | 121 (12.2) |
| total | 234 (100.0) | 754 (100.0) | 988 (100.0) |

Data are expressed as n (%). MCD, minimal change disease; FSGS, focal segmental glomerulosclerosis; MN, membranous nephropathy; MPGN, membranoproliferative glomerulonephritis; IgAN, IgA nephropathy; IgAVas, IgA vasculitis; AAV, antineutrophil cytoplasmic antibody-associated vasculitis; anti-GBMGN, anti-glomerular basement membrane glomerulonephritis; LN, lupus nephritis; DMN, diabetic nephropathy; NSc, nephrosclerosis; ATN, acute tubular necrosis; TIN, tubulointerstitial nephritis; ALPO, Alport syndrome; IRGN, infection-related glomerulonephritis; TMA, thrombotic microangiopathy; TBMD, thin basement membrane disease; AMYL amyloidosis

**Supplementary Table 5. Distribution of renal biopsy diagnoses of nephrotic syndrome by different age groups**

| Diagnosis | Nephrotic syndrome | | | | | | | | | | |
| --- | --- | --- | --- | --- | --- | --- | --- | --- | --- | --- | --- |
|  | 0–4 years | 5–9 years | 10–19 years | 20–29 years | 30–39 years | 40–49 years | 50–59 years | 60–69 years | 70–79 years | ≥ 80 years |  |
| MCD | 161 (68.8) | 128 (54.2) | 373 (63.3) | 280 (45.4) | 255 (36.0) | 243 (27.8) | 234 (19.8) | 291 (14.0) | 266 (13.1) | 94 (15.5) |  |
| FSGS | 27 (11.5) | 18 (7.6) | 48 (8.1) | 55 (8.9) | 46 (6.5) | 61 (7.0) | 63 (5.3) | 114 (5.5) | 94 (4.6) | 36 (5.9) |  |
| MN | 1 (0.4) | 4 (1.7) | 12 (2.0) | 21 (3.4) | 54 (7.6) | 113 (12.9) | 239 (20.2) | 645 (31.1) | 697 (34.2) | 170 (28.0) |  |
| MPGN | 1 (0.4) | 2 (0.8) | 10 (1.7) | 6 (1.0) | 10 (1.4) | 9 (1.0) | 23 (1.9) | 49 (2.4) | 50 (2.5) | 21 (3.5) |  |
| IgAN | 7 (3.0) | 21 (8.9) | 20 (3.4) | 53 (8.6) | 59 (8.3) | 51 (5.8) | 67 (5.7) | 110 (5.3) | 111 (5.4) | 34 (5.6) |  |
| IgAVas | 5 (2.1) | 24 (10.2) | 9 (1.5) | 12 (1.9) | 7 (1.0) | 12 (1.4) | 21 (1.8) | 40 (1.9) | 39 (1.9) | 7 (1.2) |  |
| AAV/anti-GBMGN | 0 (0.0) | 0 (0.0) | 2 (0.3) | 3 (0.5) | 4 (0.6) | 6 (0.7) | 27 (2.3) | 43 (2.1) | 49 (2.4) | 15 (2.5) |  |
| LN | 1 (0.4) | 0 (0.0) | 41 (7.0) | 99 (16.0) | 123 (17.3) | 111 (12.7) | 78 (6.6) | 73 (3.5) | 35 (1.7) | 6 (1.0) |  |
| DMN | 0 (0.0) | 0 (0.0) | 0 (0.0) | 11 (1.8) | 53 (7.5) | 123 (14.1) | 183 (15.5) | 266 (12.8) | 194 (9.5) | 45 (7.4) |  |
| NSc | 0 (0.0) | 0 (0.0) | 2 (0.3) | 3 (0.5) | 4 (0.6) | 6 (0.7) | 11 (0.9) | 22 (1.1) | 33 (1.6) | 18 (3.0) |  |
| ATN/TIN | 0 (0.0) | 1 (0.4) | 1 (0.2) | 1 (0.2) | 2 (0.3) | 1 (0.1) | 5 (0.4) | 20 (1.0) | 6 (0.3) | 2 (0.3) |  |
| ALPO | 0 (0.0) | 1 (0.4) | 4 (0.7) | 5 (0.8) | 1 (0.1) | 1 (0.1) | 0 (0.0) | 0 (0.0) | 1 (0.0) | 0 (0.0) |  |
| IRGN | 0 (0.0) | 2 (0.8) | 4 (0.7) | 10 (1.6) | 7 (1.0) | 12 (1.4) | 13 (1.1) | 33 (1.6) | 32 (1.6) | 14 (2.3) |  |
| TMA | 0 (0.0) | 0 (0.0) | 0 (0.0) | 1 (0.2) | 6 (0.8) | 0 (0.0) | 2 (0.2) | 6 (0.3) | 5 (0.2) | 1 (0.2) |  |
| TBMD | 0 (0.0) | 0 (0.0) | 0 (0.0) | 0 (0.0) | 0 (0.0) | 2 (0.2) | 1 (0.1) | 1 (0.0) | 0 (0.0) | 0 (0.0) |  |
| AMYL | 0 (0.0) | 0 (0.0) | 0 (0.0) | 0 (0.0) | 2 (0.3) | 21 (2.4) | 43 (3.6) | 85 (4.1) | 120 (5.9) | 35 (5.8) |  |
| others | 31 (13.2) | 35 (14.8) | 63 (10.7) | 57 (9.2) | 76 (10.7) | 101 (11.6) | 172 (14.6) | 277 (13.3) | 306 (15.0) | 109 (18.0) |  |
| total | 234 (100.0) | 236 (100.0) | 589 (100.0) | 617 (100.0) | 709 (100.0) | 873 (100.0) | 1,182 (100.0) | 2,075 (100.0) | 2,038 (100.0) | 607 (100.0) |  |

Data are expressed as n (%). MCD, minimal change disease; FSGS, focal segmental glomerulosclerosis; MN, membranous nephropathy; MPGN, membranoproliferative glomerulonephritis; IgAN, IgA nephropathy; IgAVas, IgA vasculitis; AAV, antineutrophil cytoplasmic antibody-associated vasculitis; anti-GBMGN, anti-glomerular basement membrane glomerulonephritis; LN, lupus nephritis; DMN, diabetic nephropathy; NSc, nephrosclerosis; ATN, acute tubular necrosis; TIN, tubulointerstitial nephritis; ALPO, Alport syndrome; IRGN, infection-related glomerulonephritis; TMA, thrombotic microangiopathy; TBMD, thin basement membrane disease; AMYL, amyloidosis

**Supplementary Table 6 A. Transitions of diagnoses over 10 years in adult patients**

| Diagnosis | –2008 | 2009–2010 | 2011–2012 | 2013–2014 | 2015–2016 | 2017 |  |
| --- | --- | --- | --- | --- | --- | --- | --- |
| MCD | 48 (4.6) | 388 (6.0) | 367 (5.8) | 358 (5.8) | 393 (6.4) | 151 (5.9) |  |
| FSGS | 42 (4.0) | 232 (3.6) | 224 (3.5) | 186 (3.0) | 200 (3.3) | 89 (3.5) |  |
| MN | 81 (7.8) | 585 (9.0) | 609 (9.6) | 602 (9.7) | 581 (9.5) | 208 (8.2) |  |
| MPGN ^†, ‡^ | 13 (1.2) | 85 (1.3) | 56 (0.9) | 71 (1.1) | 46 (0.8) | 19 (0.7) |  |
| IgAN ^†, ‡, #^ | 393 (37.7) | 2,026 (31.3) | 2,043 (32.2) | 1,963 (31.7) | 1,751 (28.6) | 662 (26.0) |  |
| IgAVas ^†, ‡^ | 13 (1.2) | 139 (2.1) | 175 (2.8) | 162 (2.6) | 175 (2.9) | 66 (2.6) |  |
| AAV/anti-GBMGN | 33 (3.2) | 373 (5.8) | 399 (6.3) | 395 (6.4) | 360 (5.9) | 158 (6.2) |  |
| LN | 34 (3.3) | 360 (5.6) | 324 (5.1) | 307 (5.0) | 276 (4.5) | 115 (4.5) |  |
| DMN ^†^ | 65 (6.2) | 372 (5.7) | 361 (5.7) | 373 (6.0) | 406 (6.6) | 177 (6.9) |  |
| NSc | 38 (3.6) | 295 (4.6) | 369 (5.8) | 321 (5.2) | 299 (4.9) | 131 (5.1) |  |
| ATN/TIN | 36 (3.5) | 209 (3.2) | 226 (3.6) | 223 (3.6) | 247 (4.0) | 90 (3.5) |  |
| ALPO | 6 (0.6) | 14 (0.2) | 13 (0.2) | 11 (0.2) | 21 (0.3) | 5 (0.2) |  |
| IRGN | 11 (1.1) | 56 (0.9) | 72 (1.1) | 75 (1.2) | 65 (1.1) | 25 (1.0) |  |
| TMA ^†, ‡^ | 4 (0.4) | 16 (0.2) | 22 (0.3) | 29 (0.5) | 35 (0.6) | 14 (0.5) |  |
| TBMD ^†, ‡^ | 5 (0.5) | 56 (0.9) | 52 (0.8) | 58 (0.9) | 102 (1.7) | 32 (1.3) |  |
| AMYL | 16 (1.5) | 102 (1.6) | 90 (1.4) | 92 (1.5) | 87 (1.4) | 37 (1.5) |  |
| others ^†, ‡^ | 204 (19.6) | 1,167 (18.0) | 949 (14.9) | 964 (15.6) | 1,078 (17.6) | 569 (22.3) |  |
| total | 1,042 (100.0) | 6,475 (100.0) | 6,351 (100.0) | 6,190 (100.0) | 6,122 (100.0) | 2,548 (100.0) |  |

Note: data are expressed n (%). MCD minimal change disease; FSGS, focal segmental glomerulosclerosis; MN, membranous glomerulonephritis; MPGN, membranoproliferative glomerulonephritis; IgAN, IgA nephropathy; IgAVas, IgA vasculitis; AAV, antineutrophil cytoplasmic antibody associated vasculitis; anti-GBMGN, anti-glomerular basement membrane glomerulonephritis; LN, lupus nephropathy; DMN, diabetic mellitus nephropathy; NSc, nephrosclerosis; ATN, acute tubular necrosis; TIN, tubulointerstitial nephritis; ALPO, Alport syndrome; IRGN, infection related glomerulonephritis; TMA, thrombotic microangiopathy; TBMD, thin basement membrane disease; AMYL, amyloidosis

† Cochran–Armitage test for trend, p<0.05.

‡ Logistic regression analysis, p<0.05 for time (year) of renal biopsy adjusted for sex, age and interaction terms.

# Logistic regression analysis, p<0.05 for age-by-time (year) of renal biopsy.

**Supplementary Table 6 B. Transitions of diagnoses over 10 years in paediatric patients**

| Diagnosis | –2008 | 2009–2010 | 2011–2012 | 2013–2014 | 2015–2016 | 2017 |
| --- | --- | --- | --- | --- | --- | --- |
| MCD | 29 (15.4) | 157 (19.3) | 138 (16.8) | 108 (15.7) | 125 (17.9) | 63 (19.7) |
| FSGS ^†, ‡^ | 14 (7.4) | 30 (3.7) | 31 (3.8) | 22 (3.2) | 26 (3.7) | 6 (1.9) |
| MN | 2 (1.1) | 14 (1.7) | 17 (2.1) | 12 (1.7) | 16 (2.3) | 10 (3.1) |
| MPGN | 0 (0.0) | 23 (2.8) | 10 (1.2) | 13 (1.9) | 6 (0.9) | 3 (0.9) |
| IgAN | 73 (38.8) | 297 (36.4) | 296 (36.1) | 246 (35.9) | 261 (37.4) | 100 (31.3) |
| IgAVas ^†, ‡^ | 12 (6.4) | 50 (6.1) | 66 (8.0) | 71 (10.3) | 66 (9.5) | 37 (11.6) |
| AAV/anti-GBMGN | 3 (1.6) | 6 (0.7) | 9 (1.1) | 5 (0.7) | 5 (0.7) | 3 (0.9) |
| LN | 6 (3.2) | 32 (3.9) | 51 (6.2) | 38 (5.5) | 20 (2.9) | 13 (4.1) |
| DMN | 0 (0.0) | 2 (0.2) | 0 (0.0) | 0 (0.0) | 0 (0.0) | 0 (0.0) |
| NSc | 0 (0.0) | 3 (0.4) | 1 (0.1) | 2 (0.3) | 1 (0.1) | 0 (0.0) |
| ATN/TIN | 3 (1.6) | 6 (0.7) | 18 (2.2) | 13 (1.9) | 11 (1.6) | 7 (2.2) |
| ALPO | 6 (3.2) | 9 (1.1) | 21 (2.6) | 10 (1.5) | 10 (1.4) | 7 (2.2) |
| IRGN ^†, ‡^ | 0 (0.0) | 4 (0.5) | 7 (0.9) | 4 (0.6) | 12 (1.7) | 3 (0.9) |
| TMA | 0 (0.0) | 1 (0.1) | 3 (0.4) | 3 (0.4) | 2 (0.3) | 1 (0.3) |
| TBMD | 2 (1.1) | 14 (1.7) | 12 (1.5) | 6 (0.9) | 11 (1.6) | 0 (0.0) |
| AMYL | 0 (0.0) | 0 (0.0) | 0 (0.0) | 1 (0.1) | 1 (0.1) | 0 (0.0) |
| others | 38 (20.2) | 167 (20.5) | 140 (17.1) | 132 (19.2) | 124 (17.8) | 67 (20.9) |
| total | 188 (100.0) | 815 (100.0) | 820 (100.0) | 686 (100.0) | 697 (100.0) | 320 (100.0) |

Note: data are expressed n (%). MCD minimal change disease; FSGS, focal segmental glomerulosclerosis; MN, membranous glomerulonephritis; MPGN, membranoproliferative glomerulonephritis; IgAN, IgA nephropathy; IgAVas, IgA vasculitis; AAV, antineutrophil cytoplasmic antibody associated vasculitis; anti-GBMGN, anti-glomerular basement membrane glomerulonephritis; LN, lupus nephropathy; DMN, diabetic mellitus nephropathy; NSc, nephrosclerosis; ATN, acute tubular necrosis; TIN, tubulointerstitial nephritis; ALPO, Alport syndrome; IRGN, infection related glomerulonephritis; TMA, thrombotic microangiopathy; TBMD, thin basement membrane disease; AMYL, amyloidosis

† Cochran–Armitage test for trend, p<0.05.

‡ Logistic regression analysis, p<0.05 for time (year) of renal biopsy adjusted for sex, age and interaction terms.

**Supplementary Table 7. Distribution of renal biopsy diagnoses of nephritic syndrome**

| Diagnosis | Nephritic syndrome | |
| --- | --- | --- |
|  | Paediatric patients (< 19 years) | Adult patients (≥ 19 years) |
| MCD | 6 (1.0) | 67 (0.8) |
| FSGS | 4 (0.7) | 174 (2.0) |
| MN | 8 (1.4) | 321 (3.8) |
| MPGN | 9 (1.5) | 59 (0.7) |
| IgAN | 375 (63.6) | 3,663 (43.1) |
| IgAVas | 38 (6.4) | 254 (3.0) |
| AAV/anti-GBMGN | 4 (0.7) | 681 (8.0) |
| LN | 17 (2.9) | 311 (3.7) |
| DMN | 0 (0.0) | 277 (3.3) |
| NSc | 2 (0.3) | 515 (6.1) |
| ATN/TIN | 8 (1.4) | 435 (5.1) |
| ALPO | 14 (2.4) | 26 (0.3) |
| IRGN | 3 (0.5) | 87 (1.0) |
| TMA | 0 (0.0) | 30 (0.4) |
| TBMD | 12 (2.0) | 131 (1.5) |
| AMYL | 0 (0.0) | 55 (0.6) |
| others | 90 (15.3) | 1,412 (16.6) |
| total | 590 (100.0) | 8,498 (100.0) |

Data are expressed as n (%). MCD, minimal change disease; FSGS, focal segmental glomerulosclerosis; MN, membranous nephropathy; MPGN, membranoproliferative glomerulonephritis; IgAN, IgA nephropathy; IgAVas, IgA vasculitis; AAV, antineutrophil cytoplasmic antibody-associated vasculitis; anti-GBMGN, anti-glomerular basement membrane glomerulonephritis; LN, lupus nephritis; DMN, diabetic nephropathy; NSc, nephrosclerosis; ATN, acute tubular necrosis; TIN, tubulointerstitial nephritis; ALPO, Alport syndrome; IRGN, infection-related glomerulonephritis; TMA, thrombotic microangiopathy; TBMD, thin basement membrane disease; AMYL, amyloidosis

**Supplementary Figure 1A.** Flow of patient selection in the present study. J-RBR, Japan Renal Biopsy Registry; J-KDR, Japan Kidney Disease Registry

**Supplemental FIGURE 1B.** Annual number of renal biopsies registered to J-RBR/J-KDR

J-RBR, Japan Renal Biopsy Registry; J-KDR, Japan Kidney Disease Registry

**Supplementary Figure 2.** The frequency of total diagnoses by different age groups.

Percentages are given only for diseases with a high frequency and are rounded to the nearest whole number.

MCD, minimal change disease; FSGS, focal segmental glomerulosclerosis; MN, membranous nephropathy; MPGN, membranoproliferative glomerulonephritis; IgAN, IgA nephropathy; IgAVas, IgA vasculitis; AAV, antineutrophil cytoplasmic antibody-associated vasculitis; anti-GBMGN, anti-glomerular basement membrane glomerulonephritis; LN, lupus nephritis; DMN, diabetic nephropathy; NSc, nephrosclerosis; ATN, acute tubular necrosis; TIN, tubulointerstitial nephritis; ALPO, Alport syndrome; IRGN, infection-related glomerulonephritis; TMA, thrombotic microangiopathy; TBMD, thin basement membrane disease; AMYL, amyloidosis

**Supplementary Figure 3.** The frequency of diagnoses with nephrotic syndrome by different age groups.

Percentages are given only for diseases with a high frequency and are rounded to the nearest whole number.

MCD, minimal change disease; FSGS, focal segmental glomerulosclerosis; MN, membranous nephropathy; MPGN, membranoproliferative glomerulonephritis; IgAN, IgA nephropathy; IgAVas, IgA vasculitis; AAV, antineutrophil cytoplasmic antibody-associated vasculitis; anti-GBMGN, anti-glomerular basement membrane glomerulonephritis; LN, lupus nephritis; DMN, diabetic nephropathy; NSc, nephrosclerosis; ATN, acute tubular necrosis; TIN, tubulointerstitial nephritis; ALPO, Alport syndrome; IRGN, infection-related glomerulonephritis; TMA, thrombotic microangiopathy; TBMD, thin basement membrane disease; AMYL, amyloidosis

**Supplementary Figure 4.** The distribution of urine abnormalities over 10 years.

Haematuria is defined as having ≥5 red blood cells (RBCs) per high-power field. Proteinuria is defined as having a urine protein level ≥0.3 g per day for adults and ≥0.15 g per day for paediatric patients.

p<0.05 by Cochran–Armitage test for trend and logistic regression analysis in all groups except for proteinuria in paediatrics patients.

**Supplementary Figure 5.** The distribution of age categories for a period of 10 years**.**

**Supplemental Note. The Investigators and Institutions Participating in the Japan Renal Biopsy Registry**

The following investigators and initial institutions have participated in the development of the J-RBR since 2007: Hirofumi Makino and Hitoshi Sugiyama (Okayama University), Takashi Taguchi (Nagasaki University), Hitoshi Yokoyama (Kanazawa Medical University), Hiroshi Sato (Tohoku University; present institution: JR Sendai Hospital), Takao Saito (Fukuoka University; present institution: Sanko Clinic), Yoshie Sasatomi (Fukuoka University; present institution: Kanenokuma Hospital), Yukimasa Kohda (Kumamoto University; present institution: Hikarinomori Clinic), Shinichi Nishi (Niigata University; present institution: Kobe University), Kazuhiko Tsuruya (Kyushu University; present institution: Nara Medical University), Yutaka Kiyohara (Kyushu University; present institution: Hisayama Research Institute for Lifestyle Diseases), Hideyasu Kiyomoto (Kagawa University; present institution: Tohoku Medical Megabank Organization, Tohoku University), Hiroyuki Iida (Toyama Prefectural Central Hospital; present institution: Toyama Prefectural Rehabilitation Hospital), Tamaki Sasaki (Kawasaki Medical School), Makoto Higuchi (Shinshu University), Motoshi Hattori (Tokyo Women’s Medical University), Kazumasa Oka (Osaka Kaisei Hospital; present institution: Hyogo Prefectural Nishinomiya Hospital), Shoji Kagami (The University of Tokushima Graduate School), Michio Nagata (University of Tsukuba), Tetsuya Kawamura (The Jikei University School of Medicine), Masataka Honda (Tokyo Metropolitan Children’s Medical Center), Yuichiro Fukasawa (KKR Sapporo Medical Center; present institution: Sapporo City General Hospital), Atsushi Fukatsu (Kyoto University Graduate School of Medicine; present institution: Fukatsu Medical Clinic), Kunio Morozumi (Japanese Red Cross Nagoya Daini Hospital; present institution: Masuko Memorial Hospital), Norishige Yoshikawa (Wakayama Medical University), Yukio Yuzawa (Fujita Health University), Seiichi Matsuo (Nagoya University), and Kensuke Joh (Chiba- East National Hospital; present institution: The Jikei University School of Medicine).

*Hokkaido District*

1. Asahikawa Medical University Hospital (Division of Cardiology, Nephrology, Pulmonology and Neurology, Department of Internal Medicine), Naoyuki Hasebe, Naoki Nakagawa
2. National Hospital Organization Hokkaido Medical Center (Department of Nephrology), Sekiya Shibazaki, Tomotsune Miyamoto, Masanori Ito
3. Hokkaido University Graduate School of Medicine (Department of Rheumatology, Endocrinology and Nephrology, Faculty of Medicine and Graduate School of Medicine, Hokkaido University), Saori Nishio, Daigo Nakazawa
4. Hokkaido University Graduate School of Medicine (Department of Pediatrics), Takayuki Okamoto, Yasuyuki Sato
5. KKR Sapporo Medical Center (Department of Pathology), Akira Suzuki
6. Sapporo Medical University (Department of Cardiovascular, Renal and Metabolic Medicine), Norihito Moniwa, Marenao Tanaka
7. Sapporo City General Hospital, Yuichiro Fukasawa
8. Teine Keijinkai Hospital (Department of Nephrology), Hideki Takizawa

*Tohoku District*

1. Iwate Prefectural Central Hospital (Department of Nephrology and Rheumatology), Jun Soma, Izaya Nakaya
2. Fukushima Medical University (Department of Nephrology and Hypertension), Junichiro James Kazama, Kennichi Tanaka, Mizuko Tanaka
3. Japan Community Health Care Organization Sendai Hospital (Department of Nephrology), Toshinobu Sato, Satoru Sanada, Hideaki Hashimoto
4. Tohoku University Hospital and affiliated hospitals (Internal Medicine), Mariko Miyazaki, Tasuku Nagasawa, Koji Okamoto
5. Yamagata University School of Medicine (Department of Cardiology, Pulmonology, and Nephrology), Tsuneo Konta, Kazunobu Ichikawa
6. Yamagata University School of Medicine (Department of Pediatrics), Daisuke Ogino

*Kanto District*

1. National Hospital Organization Chibahigashi National Hospital (Department of Pathology), Hiroshi Kitamura, (Department of Nephrology), Toshiyuki Imasawa, (Department of Pediatrics), Chieko Matsumura, (Department of Surgery), Naotake Akutsu
2. National Hospital Organization Chiba-East Hospital (Department of Urology), Koichi Kamura (*) *present address, Harunclinic Sakura
3. Dokkyo Medical University Saitama Medical Center (Department of Nephrology), Tetsuro Takeda
4. Dokkyo Medical University (Department of Nephrology and Hypertension), Toshihiko Ishimitsu
5. Gunma University Graduate School of Medicine (Department of Nephrology and Rheumatology), Keiju Hiromura, Yoriaki Kaneko, Hidekazu Ikeuchi, Toru Sakairi
6. Jichi Medical University (Division of Nephrology), Daisuke Nagata, Osamu Saito, Tetsu Akimoto, Akito Maeshima
7. The Jikei University School of Medicine (Division of Nephrology and Hypertension) Takashi Yokoo, Nobuo Tsuboi, Kentaro Koike
8. The Jikei University School of Medicine, Katsushika Medical Center (Division of Nephrology and Hypertension), Yudo Tanno, Shinya Yokote
9. The Jikei University School of Medicine, Daisan Hospital (Division of Nephrology and Hypertension), Yoichi Miyazaki, Hiroyuki Ueda, Masahiro Okabe, Mai Tanaka
10. The Jikei University Kashiwa Hospital (Division of Nephrology and Hypertension), Masato Ikeda, Akihiro Shimizu
11. Juntendo University Faculty of Medicine (Department of Nephrology), Yusuke Suzuki, Tomohito Goda, Masao Kihara, Miyuki Takagi
12. Kawaguchi Municipal Medical Center (Division of Nephrology), Masahiro Ishikawa
13. Kyorin University School of Medicine (Department of Nephrology and Rheumatology), Shinya Kaname, Miho Karube, Kazuhito Fukuoka, Takahisa Kawakami
14. Mito Saiseikai General Hospital (Division of Nephrology), Itaru Ebihara, Chihiro Sato
15. Nippon Medical School (Division of Nephrology, Department of Internal Medicine), Yukinao Sakai, Akio Hirama, Akiko Mii
16. Nihon University School of Medicine (Division of Nephrology, Hypertension and Endocrinology), Yoshinobu Fuke, Masanori Abe
17. Saitama Medical University, Faculty of Medicine (Department of Nephrology), Hirokazu Okada, Tsutomu Inoue
18. Saitama Medical University, Saitama Medical Center (Department of Nephrology and Hypertension), Takatsugu Iwashita, Yuta Kogure, Koichi Kanozawa, Hajime Hasegawa
19. Saiyu Soka Hospital (Department of Internal Medicine), Masamitsu Ubukata
20. Showa University School of Medicine (Division of Nephrology, Department of Medicine), Takanori Shibata
21. Showa University Fujigaoka Hospital (Division of Nephrology, Department of Medicine), Yoshihiko Inoue
22. St. Marianna University School of Medicine (Division of Nephrology and Hypertension, Department of Internal Medicine), Tomo Suzuki, Daisuke Ichikawa, Sayuri Shirai, Yugo Shibagaki
23. Tokai University School of Medicine (Division of Nephrology, Endocrinology and Metabolism), Takehiko Wada, Masafumi Fukagawa
24. Teikyo University School of Medicine (Department of Internal Medicine), Yoshihide Fujigaki
25. Teikyo University School of Medicine (Department of Urology), Shigeo Horie (*), Satoru Muto (*) *present address, Juntendo University School of Medicine (Department of Urology)
26. Tokyo Medical University Ibaraki Medical Center (Department of Nephrology), Masaki Kobayashi, Kouichi Hirayama, Homare Shimohata
27. Tokyo Metropolitan Children's Medical Center (Department of Nephrology), Riku Hamada (Department of General Pediatrics), Hiroshi Hataya
28. Tokyo Women's Medical University (Department of Pediatric Nephrology), Motoshi Hattori, Kenichiro Miura, Kiyonobu Ishizuka, Naoto Kaneko
29. Tokyo Women's Medical University (Department of Nephrology), Kosaku Nitta, Keiko Uchida, Takahito Moriyama
30. Toranomon Hospital, Nephrology Center, Yoshifumi Ubara, Tatsuya Suwabe, Junichi Hoshino, Noriko Hayami
31. The University of Tokyo (Department of Nephrology and Endocrinology), Masaomi Nangaku, Tetsuhiro Tanaka, Yoshifumi Hamasaki, Kenjiro Honda
32. The University of Tokyo (Department of Pediatrics), Yutaka Harita, Shoichiro Kanda, Yuko Kajiho
33. University of Tsukuba (Department of Nephrology), Kunihiro Yamagata, Joichi Usui, Tetsuya Kawamura
34. Yokohama City University Graduate School of Medicine (Department of Medical Science and Cardiorenal Medicine), Kouichi Tamura, Hiromichi Wakui, Tomohiko Kanaoka, Ryu Kobayashi
35. Yokohama City University Medical Center, Nobuhito Hirawa, Sanae Saka, Akira Fujiwara

*Koushinetsu District*

1. Niigata University Graduate School of Medical and Dental Sciences (Division of Clinical Nephrology and Rheumatology), Ichiei Narita, Shin Goto, Yumi Itoh, Naofumi Imai
2. Shinshu University School of Medicine (Department of Nephrology), Yuji Kamijo, Koji Hashimoto, Akinori Yamaguchi, Kosuke Sonoda
3. University of Yamanashi Hospital (Third Department of Internal Medicine), Kazuya Takahashi, Fumihiko Furuya

*Hokuriku District*

1. National Hospital Organization Kanazawa Medical Center (Department of Nephrology and Rheumatology), Kiyoki Kitagawa
2. Kanazawa Medical University School of Medicine (Department of Nephrology), Hitoshi Yokoyama, Keiji Fujimoto, Norifumi Hayashi
3. Kanazawa Medical University (Department of Diabetology & Endocrinology), Daisuke Koya, Munehiro Kitada, Yuka Kuroshima
4. Kanazawa University Hospital (Division of Nephrology), Takashi Wada, Kengo Furuichi, Miho Shimizu, Norihiko Sakai
5. Komatsu Sophia Hospital, Yasuhiro Katou, Yuta Yamamura
6. Koshino Internal Medicine Clinic, Yoshitaka Koshino
7. Pubulic Central Hospital of Matto-Ishikawa, Chikako Takaeda
8. Sugita Genpaku Memorial Obama Municipal Hospital, Haruyoshi Yoshida, Takayasu Horiguchi
9. Toyama Prefectural Central Hospital (Departent of Internal Medicine), Yasuyuki Shinozaki, Masahiko Kawabata
10. Toyama City Hospital (Department of Internal Medicine), Satoshi Ota, Yoh-ichi Ishida
11. University of Fukui, Faculty of Medical Sciences (Department of Nephrology), Masayuki Iwano, Naoki Takahashi, Kenji Kasuno, Daisuke Mikami
12. University of Toyama (Second Department of Internal Medicine), Hidenori Yamazaki

*Tokai District*

1. Aichi Children's Health and Medical Center (Department of Pediatric Nephrology), Naoya Fujita, Satoshi Hibino, Kazuki Tanaka
2. Aichi Medical University School of Medicine (Division of Nephrology and Rheumatology), Yasuhiko Ito, Takuhito Nagai, Takayuki Katsuno, Hironobu Nobata
3. Chuno Kosei Hospital, Shogo Kimura, Yuka Soga
4. Fujinomiya City General Hospital, Masanori Sakakima
5. Fujita Health University School of Medicine (Department of Nephrology), Yukio Yuzawa, Naotake Tsuboi, Hiroki Hayashi, Kazuo Takahashi
6. Hamamatsu University School of Medicine, University Hospital (Internal Medicine1, Division of Nephrology), Hideo Yasuda, Naro Ohashi, Taichi Sato
7. Japanese Red Cross Nagoya Daini Hospital (Kidney Center), Asami Takeda, Yasuhiro Otsuka
8. Nagoya City East Medical Center, Minamo Ono, Tatsuya Tomonari
9. Nagoya City University Graduate School of Medical Sciences (Department of Cardio-Renal Medicine and Hypertension), Michio Fukuda, Masashi Mizuno, Taisei Suzuki, Satoru Kominato
10. Nagoya Kyoritsu Hospital (Department of Internal Medicine), Hirotake Kasuga
11. Nagoya University Graduate School of Medicine (Department of Nephrology), Shoichi Maruyama, Yoshinari Yasuda, Tomoki Kosugi, Takuji Ishimoto
12. Shizuoka General Hospital (Department of Nephrology), Noriko Mori, Satoshi Tanaka
13. Mie University Graduate School of Medicine (Department of Cardiology and Nephrology), Tomohiro Murata, Mika Fujimoto, Kan Katayama
14. Japan Community Health Care Organization Yokkaichi Hazu Medeical Center (Division of Nephrology and Blood Purification), Yasuhide Mizutani, Hitoshi Kodera, Masato Miyake

*Kinki District*

1. Hyogo Prefectural Nisihinomiya Hospital (Department of Pathology), Kazumasa Oka
2. Ikeda City Hospital (Department of Nephrology), Nobuyuki Kajiwara
3. Kitano Hospital, Tazuke Kofukai Medical Research Institute (Department of Nephrology and Dialysis), Tatsuo Tsukamoto, Tomomi Endo, Eri Muso
4. Kobe University Graduate School of Medicine (Division of Nephrology and Kidney Center), Shinichi Nishi, Shunsuke Goto
5. Kobe University Graduate School of Medicine (Department of Pediatrics), Kazumoto Iijima, Kandai Nozu, Tomohiko Yamamura
6. Japan Community Health Care Organization Kobe Central Hospital, Yoko Adachi, Takaaki Nishihara, Michitsugu Kamezaki
7. National Hospital Organization Kyoto Medical Center (Division of Nephrology), Koichi Seta, Kensei Yahata
8. Kyoto Prefectural University of Medicine Graduate School of Medical Science (Department of Nephrology), Keiichi Tamagaki, Tetsuro Kusaba, Yayoi Shiotsu
9. Kyoto University Graduate School of Medicine (Department of Nephrology), Motoko Yanagita, Hideki Yokoi, Kaoru Sakai, Akira Ishii
10. Nara Medical University (Department of Nephrology), Kazuhiko Tsuruya, Kenichi Samejima
11. National Cerebral and Cardiovascular Center (Division of Hypertension and Nephrology), Fumiki Yoshihara
12. Osaka City University Graduate School of Medicine (Department of Nephrology), Katsuhito Mori, Akihiro Tsuda, Shinya Nakatani
13. Osaka City General Hospital (Division of Nephrology and Hypertension), Yoshio Konishi, Takashi Morikawa, Chizuko Kitabayashi
14. Osaka City General Hospital (Division of Pediatrics), Rika Fujimaru
15. Osaka General Medical Center (Department of Kidney Disease and Hypertension), Terumasa Hayashi, Tatsuya Shoji
16. Osaka Women's and Children's Hospital (Department of Pediatric Nephrology and Metabolism), Katsusuke Yamamoto
17. Osaka Medical College (Department of Pediatrics), Akira Ashida
18. Osaka Red Cross Hospital (Department of Nephrology), Akira Sugawara, Masao Koshikawa, Yoshihisa Ogawa, Tomoko Kawanishi
19. Osaka Rosai Hospital (Department of Nephrology), Atsushi Yamauchi, Katsuyuki Nagatoya, Daisuke Mori, Ryota Haga
20. Osaka University Graduate School of Medicine (Department of Nephrology), Yoshitaka Isaka, Ryohei Yamamoto, Tomoko Namba
21. Saiseikai Shiga Hospital (Division of Nephrology), Toshiki Nishio
22. Shiga University of Medical Science (Department of Medicine), Shinichi Araki
23. Shirasagi Hospital (Kidney Center), Shigeichi Shoji, Kenjiro Yamakawa, Senji Okuno
24. Toyonaka Municipal Hospital (Division of Nephrology), Megumu Fukunaga
25. Wakayama Medical University (Department of Pediatrics), Yuko Shima, Taketsugu Hama
26. Wakayama Medical University (Department of Nephrology), Takashi Shigematsu, Masaki Ohya

*Chugoku District*

1. Kawasaki Medical School (Department of Nephrology and Hypertension), Naoki Kashihara, Tamaki Sasaki, Hajime Nagasu
2. Kurashiki Central Hospital (Division of Nephrology), Kenichiro Asano, Motoko Kanzaki, Kosuke Fukuoka
3. Hiroshima University Hospital (Department of Nephrology), Takao Masaki, Shigehiro Doi, Ayumu Nakashima, Toshiki Doi
4. Mizushima Kyodo Hospital (Department of Nephrology), Kan Yamazaki, Nobuyoshi Sugiyama, Yuichiro Inaba, Kouji Ozeki
5. Okayama Saiseikai General Hospital (Department of Nephrology), Makoto Hiramatsu, Keisuke Maruyama, Noriya Momoki
6. Okayama University Graduate School of Medicine, Dentistry, and Pharmaceutical Sciences (Department of Nephrology, Rheumatology, Endocrinology and Metabolism), Hiroshi Morinaga, Ayu Akiyama, Natsumi Uchiyama, Mariko Nishiwaki
7. Saiseikai Yamaguchi General Hospital (Department of Internal Medicine), Tsuyoshi Imai
8. Shimane University Faculty of Medicine (Division of Nephrology), Takafumi Ito, Masahiro Egawa, Shohei Fukunaga
9. Tottori University, Faculty of Medicine (Division of Pediatrics and Perinatology), Shinichi Okada, Koichi Kitamoto, Hiroki Yokoyama, Yuko Yamada

*Shikoku District*

1. Kagawa University, Faculty of Medicine (Department of Cardiorenal and Cerebrovascular Medicine & Department of Clinical Pathology), Tadashi Sofue, Tetsuo Minamino, Emi Ibuki
2. Kochi University, Kochi Medical School (Department of Endocrinology, Metabolism and Nephrology), Yoshio Terada, Taro Horino, Yoshiko Shimamura, Tatsuki Matsumoto
3. Kochi University, Kochi Medical School (Department of Pediatrics), Mikiya Fujieda, Masayuki Ishihara
4. Tokushima University Graduate School (Department of Pediatrics, Institute of Biomedical Sciences), Shoji Kagami, Maki Urushihara, Yukiko Kinoshita
5. Tokushima University Graduate School (Department of Nephrology, Institute of Biomedical Sciences), Hideharu Abe, Kojiro Nagai

*Kyushu District*

1. Fukuoka University (Division of Nephrology and Rheumatology, Department of Internal Medicine, Faculty of Medicine), Kosuke Masutani, Tetsuhiko Yasuno, Kenji Ito
2. Japanese Red Cross Fukuoka Hospital (Department of Pediatrics), Ken Hatae, Manao Nishimura, Hiroyo Maruyama
3. Japanese Red Cross Fukuoka Hospital (Nephrology and Dialysis Center), Koji Mitsuiki
4. Kumamoto University Graduate School of Medical Sciences (Department of Nephrology), Masashi Mukoyama, Masataka Adachi
5. Kurume University School of Medicine (Division of Nephrology, Department of Medicine), Kei Fukami, Junko Yano
6. Kyushu University Graduate School of Medical Sciences (Department of Medicine and Clinical Science), Toshiaki Nakano, Akihiro Tsuchimoto, Shunsuke Yamada, Yuta Matsukuma
7. Kyushu University Graduate School of Medical Sciences (Department of Environmental Medicine), Yutaka Kiyohara, Toshiharu Ninomiya, Masaharu Nagata
8. Miyazaki Prefectural Miyazaki Hospital (Division of Nephrology), Naoko Yokota-Ikeda, Keiko Kodama
9. Nagasaki University Hospital (Department of Pathology), Takashi Taguchi
10. Nagasaki University Hospital (Department of Nephrology), Tomoya Nishino, Yoko Obata, Tadashi Uramatsu
11. National Fukuoka Higashi Medical Center (Kidney Unit), Ritsuko Katafuchi
12. National Hospital Organization Kyushu Medical Center, Masaru Nakayama
13. Oitaken Kouseiren Tsurumi Hospital (Division of Nephrology), Ryokichi Yasumori
14. Saga University, Faculty of Medicine (Department of Internal Medicine), Motoaki Miyazono, Eriko Nonaka, Shuichi Rikitake, Makoto Fukuda
15. St. Mary's Hospital, Harumichi Higashi
16. University of Miyazaki Hospital (Division of Nephrology), Shouichi Fujimoto, Yuji Sato, Masao Kikuchi, Akihiro Minakawa
17. University of Occupational and Environmental Health (Second Department of Internal Medicine), Masahito Tamura, Tetsu Miyamoto

University of the Ryukyus Graduate School of Medicine (Department of Cardiology, Nephrology and Neurology), Yusuke Ohya, Kentaro Kohagura
